# The Effect of Aging on Resting State Connectivity of Predefined Networks in the Brain

**DOI:** 10.3389/fnagi.2019.00234

**Published:** 2019-09-04

**Authors:** Eleanna Varangis, Christian G. Habeck, Qolamreza R. Razlighi, Yaakov Stern

**Affiliations:** Division of Cognitive Neuroscience, Department of Neurology, College of Physicians and Surgeons, Columbia University, New York, NY, United States

**Keywords:** aging, cognition, fMRI, functional connectivity, graph theory

## Abstract

Recent studies have found a deleterious effect of age on a wide variety of measures of functional connectivity, and some hints at a relationship between connectivity at rest and cognitive functioning. However, few studies have combined multiple functional connectivity methods, or examined them over a wide range of adult ages, to try to uncover which metrics and networks seem to be particularly sensitive to age-related decline across the adult lifespan. The present study utilized multiple resting state functional connectivity methods in a sample of adults from 20–80 years old to gain a more complete understanding of the effect of aging on network function and integrity. Whole-brain results showed that aging results in weakening average within-network connectivity, lower system segregation and local efficiency, and higher participation coefficient. Network-level results suggested that nearly every primary sensory and cognitive network faces some degree of age-related decline, including reduced within-network connectivity, higher network-based participation coefficient, and reduced network-level local efficiency. Further, some of these connectivity metrics showed relationships with cognitive performance. Thus, these results suggest that a multi-method analysis of functional connectivity data may be critical to capture the full effect of aging on the health of brain networks.

## Introduction

One commonly reported complaint in the context of healthy aging is that of cognitive decline – adults show reductions in processing speed, attentional resources, working and episodic memory, and inhibitory processing as they age (Craik and Byrd, 1982; Hasher and Zacks, 1988; Salthouse, 1996). At the same time, changes in brain structure (Peter, 1979; Kemper, 1994; Damoiseaux et al., 2009) and function (Li and Lindenberger, 1999; Cabeza, 2002; Cabeza et al., 2002) over the course of adulthood have been well-documented, and suggest that many aspects of brain health also decline throughout the adult lifespan. While some previous studies have found links between structural and functional outcomes and cognition (i.e., Miller et al., 2008; Hedden et al., 2016), recent studies have focused on how changes in patterns of correlated activity in the brain may underlie age-related cognitive decline (Campbell et al., 2012; Onoda et al., 2012; Sala-Llonch et al., 2012, 2014; Avelar-Pereira et al., 2017). In the current study, we investigated how aging is associated with integrity of and interactions among functional brain networks at rest, and how these age-related differences may be associated with cognitive outcomes.

There has recently been increasing interest in examining interactions among networks using functional connectivity techniques which model correlations among functional magnetic resonance imaging (fMRI) blood oxygen level dependent (BOLD) signals from specific regions of interest (ROIs). These studies can be divided along several lines: (1) those examining a few select networks vs. those looking more broadly across the whole brain, (2) those using seed-based techniques to capture regions connected to specific ROIs vs. those modeling connectivity across many predefined ROIs distributed throughout the brain, (3) those using a predefined network structure vs. those deriving network structure from participant-level data, and (4) those measuring connectivity at rest vs. those measuring connectivity during a cognitive or motor task. While many approaches have been taken, recent studies have focused on examining connectivity (1) across the whole brain, (2) modeling connectivity across multiple predefined ROIs, (3) deriving a network structure from participant/group level data, and (4) measuring connectivity at rest. Previous studies using this approach have found that with aging functional connectivity tends to be reduced within-network (especially in cognitive, non-sensory networks), increased between networks, and show a generally less segregated/modular network structure (Onoda et al., 2012; Tomasi and Volkow, 2012; Betzel et al., 2014; Chan et al., 2014; Sala-Llonch et al., 2014; Song et al., 2014; Geerligs et al., 2015; King et al., 2017). This corroborates past research using more exploratory, seed-based analyses (Andrews-Hanna et al., 2007; Jones et al., 2011; Campbell et al., 2012), or examining specific networks or connections between nodes (Wang et al., 2010) to uncover the effects of aging on functional connectivity. Further, many of these studies (and similar ones) found that these patterns of connectivity may be related to cognitive performance, thus potentially implicating their role in age-related cognitive decline (Andrews-Hanna et al., 2007; Wang et al., 2010; Onoda et al., 2012; Chan et al., 2014; Sala-Llonch et al., 2014; Geerligs et al., 2015; King et al., 2017). Extending these findings to whole-brain connectivity during a cognitive task, two additional studies have also found alterations in network connectivity during a task in aging (Geerligs et al., 2014; Varangis et al., 2019), suggesting that in addition to showing differential connectivity at rest, aging brains also respond differently to a cognitive challenge, and these connectivity patterns may in turn be related to cognitive performance on the in-scanner task (Varangis et al., 2019).

While these existing studies have examined many metrics of functional connectivity in older and younger adults, few have systematically compared the effect of age on several techniques for measuring functional connectivity, and fewer still have included a wide range of ages (i.e., beyond just younger vs. older adults). As such, some of these past studies may be painting an incomplete picture of the magnitude or presence of the effect of aging on functional connectivity, or they may be missing the key “missing window” of middle adulthood in examining age-related differences in these metrics. One such study by Geerligs et al. (2015) utilized both graph theory and whole-brain correlational metrics for assessing functional connectivity in younger and older adults who completed a resting state scan. While this study explored which of several metrics of functional connectivity showed effects of aging, it did not include a middle-aged group. Thus, the present study included most of the same metrics as this previous study, but also included two middle-aged groups in order to test whether any effects of age are gradual across the adult lifespan, or become exaggerated (or only appear) with increasing age.

One approach commonly used in studies examining within- and between-network connectivity involves using an independent components analysis (ICA), clustering methods, or community detection to define network membership from a set of ROIs. These analyses determine the optimal network structure by identifying sets of regions (or components) that function largely independently of other sets of regions, then cross-referencing the location of each set of regions with those of established brain networks to generate network assignments for each component. Since these analyses can either be run at the individual or group level, studies using these techniques to compare groups must either identify networks that are common to all participants, or identify networks that are common to participants within each group. The benefit of the former option is that the network structure is identical (and thus comparable) across all participants, facilitating between-group comparisons, however, the benefit of the latter option is that it generates network structures that better reflect the underlying network topology of each sub-group of participants. While many studies have used these techniques to identify network structure in older and younger adults (i.e., Chan et al., 2014; Geerligs et al., 2015), it is unclear the degree to which the functional connectivity outcomes in these studies were influenced by these internal, optimally defined, network parcellations. Further, since these parcellations are typically conducted separately for older and younger participants, comparing patterns of network connectivity between younger and older participants will likely entail comparison of non-identical networks. To address this limitation, the present study aimed to utilize an external parcellation scheme in order to determine network membership that is unbiased by participant age or the methods of the present study. Power’s network parcellations were found to be fairly stable across replication (Power et al., 2011), suggesting that these network architectures may be unbiased, valid schemas for use in external samples. Thus, all network-based functional connectivity measures in the present study were computed based on this unbiased, external schema in order to investigate the effect of participant age on these metrics.

The present study examined multiple techniques for measuring resting state functional connectivity: graph theory (Rubinov and Sporns, 2010; Sporns, 2011), the metric of system segregation (Chan et al., 2014), and an average correlation approach (i.e., Geerligs et al., 2015; King et al., 2017). Based on previous studies of functional connectivity in aging, the hypotheses of the present study were as follows: (1) older adults (OA) will show reduced local efficiency (but not global efficiency) relative to younger adults (Song et al., 2014; Geerligs et al., 2015), (2) OAs will show reduced modularity relative to younger adults (Betzel et al., 2014; Song et al., 2014; Geerligs et al., 2015), (3) OAs will show increased participation coefficient relative to younger adults (Chan et al., 2014; Geerligs et al., 2015), (4) OAs will show less segregation between networks relative to younger adults (Chan et al., 2014), (5) OAs will show reduced within-network correlations and increased between-network correlations relative to younger adults (Betzel et al., 2014; Chan et al., 2014; Geerligs et al., 2015), (6) OAs will generally show a less negative brain graph relative to younger adults (Geerligs et al., 2015; Ferreira et al., 2016), and (7) some of these metrics of resting state connectivity will be related to cognitive performance (Andrews-Hanna et al., 2007; Wang et al., 2010; Onoda et al., 2012; Chan et al., 2014; Sala-Llonch et al., 2014; Geerligs et al., 2015; King et al., 2017). While these hypotheses do not specifically address where middle-aged adults fall into these patterns, it is expected that middle-aged adults will fall somewhere between younger and older adults, with the difference between younger and older adults being the primary comparison of interest in relation to findings from previous studies. That being said, the inclusion of middle-aged adults in the present study is critical to determine whether these functional connectivity metrics show gradual changes over the adult lifespan, or if age-related differences in the metrics are primarily driven by the differences between the youngest and oldest participants.

## Materials and Methods

### Participants

The sample for the present study was comprised of participants who completed the baseline visit for two studies: the Reference Ability Neural Network (RANN) study (*N* = 426) (Stern et al., 2014), or the Cognitive Reserve (CR) study (*N* = 239; some overlapping with RANN participants). Both studies included the same inclusion/exclusion criteria, the same structural and resting state functional imaging protocols, and many of the same cognitive assessments and questionnaires; the primary difference between the two studies being the functional task-based imaging protocols used, which will not be discussed here. All participants were native English speakers, right-handed, free of MRI contraindications, and read at a fourth grade reading level or above. Screening was performed prior to enrollment in order to ensure that no participants had any psychological or medical conditions that could affect cognitive function, and that OAs did not meet criteria for dementia or MCI at baseline. In order to facilitate testing of age group effects, the variable “age” was transformed into a categorical variable reflecting the following age group delineations: young adults (YA; age 20–34, *n* = 103), younger middle-aged adults (yMA; age 35–49, *n* = 63), older middle-aged adults (oMA; age 50–64, *n* = 136), and older adults (OA; age 65–80, *n* = 146).

For inclusion in the present study, participants had to meet the following additional inclusion criteria: completion of a resting state scan, and less than 30% motion artifact data removal (scrubbing) from that resting state scan (Power et al., 2012; Parkes et al., 2018). Based on these additional criteria, the final sample was comprised of 427 (YA *n* = 101, yMA *n* = 61, oMA *n* = 126, OA *n* = 139) healthy adults between the ages of 20 and 80 who met all inclusion criteria.

### fMRI Scan Parameters

The present study collected fMRI scans during a 5- (*n* = 142) or 9.5-(*n* = 286) minute resting state protocol. All participants completed these scans on a 3.0T Philips Achieva Magnet. T1-weighted images of the whole brain were acquired for each subject with a Magnetization Prepared Rapid Gradient Echo (MPRAGE) sequence with the following parameters: TE/TR: 3/6.5 ms; Field of view: 256 mm; Flip angle: 8°; In-plane resolution: 256 × 256 voxels; Slice thickness/gap: 1/0 mm; Slices: 180. fMRI blood oxygen level-dependent (BOLD) resting state scans were collected with the following parameters: TE/TR: 20/2000 ms; Flip angle: 72°; In-plane resolution: 112 × 112 voxels; Slice thickness/gap: 3/0 mm; Slices: 37.

### fMRI Data Processing

Images were preprocessed using an in-house developed native space method (Razlighi et al., 2014) as described and utilized previously in Varangis et al. (2019). The preprocessing pipeline included slice-timing correction and motion correction performed in FSL (Jenkinson et al., 2002, 2012), calculation of frame-wise displacement (FWD; as described in Power et al., 2011), volume replacement for contaminated volumes (Carp, 2013), band-pass filtering using flsmaths–bptf (Jenkinson et al., 2012), and residualization of the processed data with respect to FWD, root mean square difference of the BOLD signal, left and right hemisphere white matter, and lateral ventricular signals (Birn et al., 2006). T1 image segmentation was performed using FreeSurfer (Dale et al., 1999; Fischl et al., 2002, 2004), and inspected visually for any possible inaccuracies. In order to perform the functional connectivity analyses described below, the coordinates of the 264 ROIs identified by Power et al. (2011) were transferred to native space via non-linear registration of the subject’s structural scan to the MNI template using the ANTS software package. Next, a 10 mm radius spherical mask was generated for each coordinate and intersected with the FreeSurfer gray matter mask in order to derive the gray matter-registered ROI masks for each of the 264 ROIs. An intermodal, intra-subject, rigid-body registration of the fMRI reference image and T1 scan was then performed using FLIRT with 6 degrees of freedom, normalized mutual information as the cost function (Jenkinson and Smith, 2001), in order to transfer ROI masks from T1 space to fMRI space. These transferred ROI masks were used to average all voxels within each mask to obtain a single fMRI time-series for each of the 264 ROIs.

**FIGURE 1:**
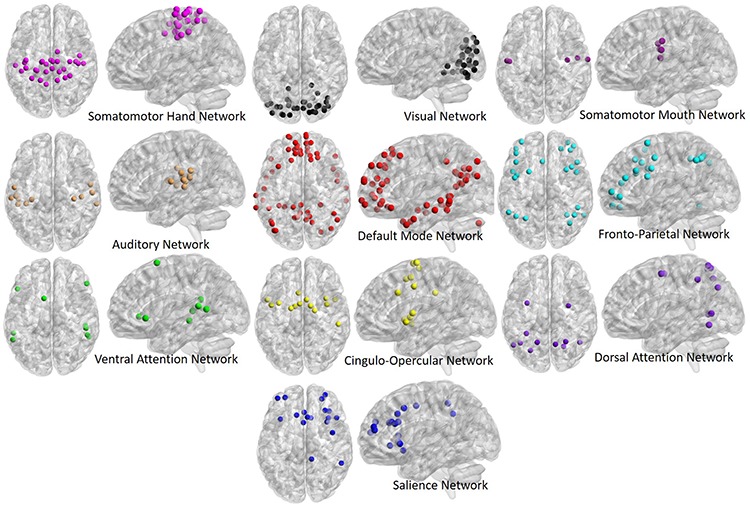
Regions of interest (ROIs) used in the present analyses, derived from the Power et al. (2011) atlas. ROIs are depicted in both their axial **(left)** and sagittal **(right)** view for each network to better illustrate spatial locations. ROIs fall into 10 primary networks, and are comprised of spherical volumes centered around coordinates, as outlined in the original atlas.

Time-series data from each ROI were used to generate correlation matrices among all ROIs (264 ROIs by 264 ROIs), and were then z-transformed to generate normalized correlation matrices for each participant. The diagonal of each correlation matrix was set to zero for all graph theory analyses, and “NA” for all average correlation analyses, in order to remove correlations between an area and itself from analyses. Additionally, ROIs with centers located within 20 mm of one another were set to “NA” as per Power et al. (2011). ROIs were then labeled based on the Power et al. (2011) network assignments, with the following networks being selected for analysis based on their inclusion in similar past studies (Chan et al., 2014; Geerligs et al., 2015; for visual depiction of all network ROIs please see Figure 1): Somatomotor Hand (Hand; 30 ROIs), Visual (Vis; 31 ROIs), Somatomotor Mouth (Mouth; 5 ROIs), Auditory (Aud; 13 ROIs), Default Mode (DMN; 58 ROIs), Salience (Sal; 18 ROIs), Cingulo-Opercular (CO; 14 ROIs), Frontoparietal (FP; 25 ROIs), Dorsal Attention (DAN; 11 ROIs), and Ventral Attention (VAN; 9 ROIs).

### Functional Connectivity Analyses

Individual z-transformed correlation matrices were used to compute several measures of functional connectivity:

#### Positive/Negative Correlation Weights

**FIGURE 2:**
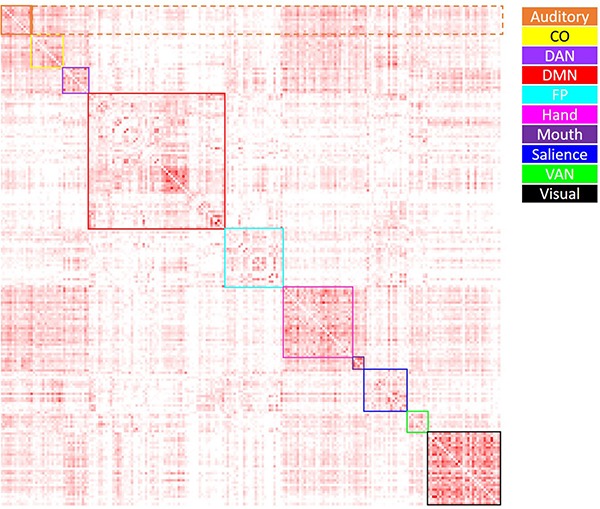
Visual depiction of the derivation of the average positive correlation metric for each network. Boxes along the diagonal reflect values averaged in order to generate the average within-network positive correlation, while values within the same row (not including the box along the diagonal) reflect values averaged to generate the average positive between-network correlation. For example, for the auditory network (depicted in orange), the values within the solid orange box were averaged to generate each participant’s average within-auditory positive correlation, while the values within the dashed orange box were averaged to generate each participant’s average between-auditory positive correlation.

**FIGURE 3:**
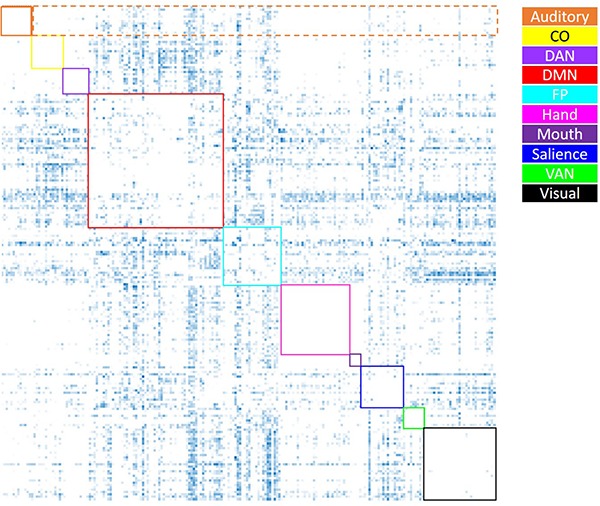
Visual depiction of the derivation of the average negative correlation metric for each network. Boxes along the diagonal reflect values that represent the average within-network negative correlation (not analyzed here), while values within the same row (not including the box along the diagonal) reflect values averaged to generate the average negative between-network correlation (the only negative correlation metric utilized). For example, for the auditory network (depicted in orange), the values within the dashed orange box were averaged to generate each participant’s average between-auditory negative correlation.

Average positive and negative correlation were computed within and between all networks of interest. Within-network correlations were characterized as those reflecting correlations between ROIs within a specific network; between-network correlations were characterized as those reflecting correlations between ROIs from one network and those of all other networks. Average positive correlation was computed by setting all negative correlation values to “NA,” then taking the average within- and between-network positive correlation for each network (see Figure 2). Average negative correlation was computed by setting all positive correlation values to “NA,” then taking the average within- and between-network negative correlation for each network (see Figure 3). Due to few negative within-network correlations (and concern as to how to interpret these values), only between-network negative correlations were included in the analysis of negative correlations. Thus, data from this analysis included the average within-network positive correlation (10 values), average between-network positive correlation (10 values), and average between-network negative correlation (10 values) for each participant. In order to examine the effect of age group on these metrics, a 4 (age group: YA, yMA, oMA, OA) × 2 (correlation direction: within, between) × 10 (network: Vis, Hand, Mouth, Aud, DMN, Sal, FP, CO, DAN, VAN) MANCOVA (covariate: scrubbing percentage) was performed for positive correlations, and a 4 (age group: YA, yMA, oMA, OA) × 10 (network: Vis, Hand, Mouth, Aud, DMN, Sal, FP, CO, DAN, VAN) MANCOVA (covariate: scrubbing percentage) was performed for negative between-network correlations. Significant interactions were probed using follow-up MANCOVA and ANOVA analyses.

These data were also used to derive the metric of system segregation introduced by Wig and colleagues (Chan et al., 2014, 2017; Wig, 2017). This metric reflects the degree to which the brain segments into networks (or systems) that function independently of one another – high values reflect greater functional separation between networks, while lower values reflect less functional separation between networks. In order to compute this metric, only positive correlations among the ten networks identified above were considered, and all negative correlations were set to zero (Chan et al., 2014, 2017). Then, the average within- and between-network correlations were calculated, and the whole-brain system segregation was defined as:

$$S\,S=\frac{{\bar{z}}_{w\,i\,t\,h\,i\,n}-{\bar{z}}_{b\,e\,t\,w\,e\,e\,n}}{{\bar{z}}_{w\,i\,t\,h\,i\,n}}$$

A one-way ANOVA was used to test whether the four age groups differed on this metric.

#### Graph Theory Metrics of Connectivity

Several graph theory metrics of functional connectivity were also computed using the Brain Connectivity Toolbox^1^ (Rubinov and Sporns, 2010) in order to measure additional aspects of global and nodal connectivity. Global connectivity was assessed using the graph theory metrics of modularity (the extent to which the correlation matrix can be partitioned into networks that maximize within-group connections and minimize between-group connections) (Newman, 2006) and global efficiency (average inverse shortest path length) (Latora and Marchiori, 2001). Nodal connectivity was assessed using the graph theory metrics of participation coefficient (using Power network partitioning – metric reflecting the number of between-network connections relative to the total number of connections at each node) (Guimera and Nunes Amaral, 2005) and local efficiency (global efficiency metric computed on the neighborhood of the node) (Latora and Marchiori, 2001). All nodal metrics were then averaged by network in order to examine any effect of network membership on these metrics. In order to ensure that results were not biased by the connectivity weight threshold applied to the correlation matrices, a range of thresholds between 2–10% (in increments of 1%) was applied to matrices during computation of each metric (i.e., Geerligs et al., 2015). Based on this thresholding, all graph theory metrics were only computed on positive correlation weights. As such, all graph theory analyses will evaluate the effects of both age group and threshold on the metric of interest. In order to assess the effect of age group on graph theory metrics, a 4 (age group: YA, yMA, oMA, OA) × 9 (threshold: 2–10%) × 10 (network) MANCOVA (covariate: scrubbing percentage) was conducted for each local metric, and a 4 (age group: YA, yMA, oMA, OA) × 9 (threshold: 2–10%) MANCOVA (covariate: scrubbing percentage) was conducted for each global metric. Significant interactions were probed using follow-up MANCOVA (age group × network at each threshold) and one-way ANOVA (effect of age group on each network for thresholds exhibiting a significant age × network interaction) analyses.

#### Continuous Effect of Age

In order to test whether age exhibited a continuous effect on these metrics, follow-up correlational analyses were performed on each metric or network that showed a main effect of age group. Thus, any global or network-based measure that showed a main effect of age was included in a correlational analysis to test for a linear effect of age on the metric (correlation between age and each metric of interest).

### Neurocognitive Assessment

Participants completed a battery of neuropsychological tasks as part of their baseline study visit. Tasks were administered in the following fixed order: Wechsler Adult Intelligence Scale (WAIS-III; Wechsler, 1997), Letter-Number Sequencing, American National Adult Reading Test (AMNART; Wechsler, 1997), Selective Reminding Task (SRT) immediate recall (Buschke and Fuld, 1974), WAIS-III Matrix Reasoning (Wechsler, 1997), SRT delayed recall and delayed recognition (Buschke and Fuld, 1974), WAIS-III Digit Symbol (Wechsler, 1997), Trail-Making Test versions A and B (TMT-A/B; Reitan, 1978), Controlled Word Association (C-F-L) and Category Fluency (animals; Benton et al., 1983), Stroop Color Word Test (Golden, 1975), Wechsler Test of Adult Reading (WTAR; Holdnack, 2001), WAIS-III Vocabulary (Wechsler, 1997), and WAIS-III Block Design (Wechsler, 1997). Based on prior analyses using these tasks in our lab, the tasks were clustered into four primary cognitive domains (Razlighi et al., 2017): Episodic Memory (all SRT outcomes), Vocabulary (WAIS Vocabulary, WTAR, AMNART), Processing Speed (WAIS Digit Symbol, Stroop Color, Stroop Color Word, TMT-A), and Fluid Reasoning (WAIS Matrix Reasoning, WAIS Block Design, TMT-B). Following collection of all baseline participant data, performance on each task was *z*-scored relative to the mean and standard deviation for each task within the whole sample of participants enrolled in the RANN and CR studies who completed these assessments. The *z*-scores for all tasks within each cognitive domain were then averaged in order to generate domain-based *z*-scores. The primary metrics used for analysis in the present study were the participant-level *z*-scores representing standardized performance in each domain.

In order to examine any relationships between out-of-scanner neuropsychological task performance and resting state connectivity metrics, Pearson correlational analyses were conducted between *z*-scored task performance in each domain and each of the connectivity metrics generated (for graph theory analyses, only thresholds that showed significant differences between age groups were used for analysis). Due to the exploratory nature of these analyses, *p*-value correction was not performed for multiple comparisons, and thus only networks that showed significant correlations with performance across multiple network-based metrics, or whole-brain metrics showing a consistent relationship with task performance will be discussed below.

## Results

### Participants

Demographic characteristics of participants in the present study are summarized in Table 1. Participant groups did not differ on education (*p* = 0.261) or gender distributions (*p* = 0.076), however, they did differ on age (*p* < 0.001), and performance on all cognitive tasks (VOCAB *p* < 0.001, SPEED *p* < 0.001, FLUID *p* < 0.001, and MEM *p* < 0.001; see Table 1). Further, the groups also differed in scrubbing percentage, such that oMAs and OAs tended to have higher scrubbing percentage than YAs. As a result, all MANCOVAs presented below include scrubbing percentage as a covariate to account for any effect of scrubbing percentage on the observed patterns of results.

**TABLE 1:** Sample demographics by age group (Young Adults, or YA; younger Middle Adults, or yMA; older Middle Adults, or oMA; Older Adults, or OA).

	**YA (*n* = 101)**	**yMA (*n* = 61)**	**oMA (*n* = 126)**	**OA (*n* = 139)**	***F* (*p*-value)**
Age	**27.604 (3.855)**	**42.393 (4.488)**	** 58.556 (4.577)**	** 70.093 (3.905)**	**2219.969 (<0.001)a**
Education	16.030 (2.324)	15.869 (2.533)	16.206 (2.033)	16.504 (2.541)	1.349 (0.258)
% Male	33.663%	49.180%	47.619%	48.921%	χ^2^ = 6.889 (0.076)
VOCAB	**−0.219 (0.907)**	**−0.386 (0.887)**	**0.164 (0.815)**	**0.315 (0.817)**	**13.449 (<0.001)b**
SPEED	**0.823 (0.701)**	**0.215 (0.629)**	**−0.108 (0.772)**	**−0.418 (0.698)**	**59.883 (<0.001)a**
FLUID	**0.688 (0.727)**	**0.140 (0.721)**	**−0.064 (0.752)**	**−0.251 (0.681)**	**36.839 (<0.001)c**
MEM	**0.687 (0.700)**	**0.435 (0.636)**	**−0.077 (0.929)**	**−0.368 (0.893)**	**34.745 (<0.001)d**
Scrubbing %	**3.092 (4.198)**	4.476 (6.063)	**6.089 (7.167)**	**6.844 (7.646)**	**7.181 (<0.001)e**

*Values reflect means (standard deviations in parentheses) for each age group. All *F*-values with *p* < 0.006 (MC-corrected) are bolded; all means representing groups that differ from one another are also bolded (a = all groups differ, b = oMA&OA > YA&yMA, c = YA > all and yMA > OA, d = YA&yMA > oMA > OA, e = YA < oMA&OA).*

### Positive and Negative Correlations

**FIGURE 4:**
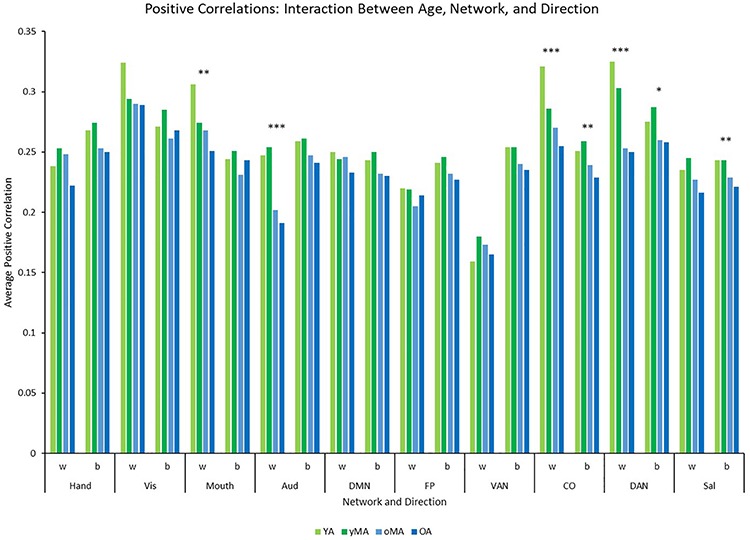
Graph depicting the interaction among age, network, and direction showing the average positive correlation for each network and direction by age group (light green = young adults, darker green = younger middle adults, light blue = older middle adults, darker blue = older adults). Significance of the main effect of age at each network/direction is represented by asterisks: ^∗∗∗^*p* ≤ 0.001, ^∗∗^*p* < 0.01, ^∗^*p* < 0.05.

A 4 (age group: YA, yMA, oMA, OA) × 2 (direction: within, between) × 10 (network) MANCOVA revealed significant effects of age group (*F*_3__,__420_ = 8.072, *p* < 0.001), and network (*F*_9__,__3780_ = 62.695, *p* < 0.001) on positive correlation weight. Additionally, interactions among direction and age group (*F*_3__,__420_ = 7.247, *p* < 0.001), network and age group (*F*_27__,__3780_ = 2.489, *p* < 0.001), direction and network (*F*_9__,__3780_ = 40.821, *p* < 0.001), and direction and network and age group (*F*_27__,__3780_ = 3.103, *p* < 0.001), and were all significant. The main effect of direction (*F*_1__,__420_ = 0.001, *p* = 0.971) was not significant. The main effect of age manifested such that YAs and yMAs showed a higher average positive correlation weight than OAs, and YAs have a higher average positive correlation than oMAs (see Table 2). Follow-up analyses probing the interaction among network and direction and age group showed an interaction between network and age group for both positive within- (*F*_27__,__3780_ = 2.914, *p* < 0.001) and between-(*F*_27__,__3798_ = 1.557, *p* = 0.033) network correlations. Analysis by network showed that there was an effect of age on within-network correlations for the Mouth, Auditory, CO, and DAN networks, and an effect of age on between-network correlations for the CO, DAN, and Sal networks (see Table 3 and Figure 4). Further, correlational analyses revealed linear effects of age on positive correlation weight in the networks mentioned above, such that higher age was associated with decreased positive within/between-network correlation strength in the above networks (see Table 3).

**TABLE 2:** Main effect of age (*F*-value and *p*-value) for each connectivity metric, means of each metric by age group, and post hoc tests for significant main effects of age (*p*-values in parentheses) among all age group combinations.

**Metric**	***F* (*p*-value)**	**Means by Age Group**				***Post Hoc* Tests**					
		**YA**	**yMA**	**oMA**	**OA**	**YA vs. yMA**	**YA vs. oMA**	**YA vs. OA**	**yMA vs. oMA**	**yMA vs. OA**	**oMA vs. OA**
Positive Correlation Weights	**8.072 (<0.001)**	0.259 (0.040)	0.258 (0.047)	0.240 (0.045)	0.234 (0.047)	0.001 (>0.999)	**0.018 (0.011)**	**0.024 (<0.001)**	*0.018 (0.052)*	**0.024 (0.003)**	0.006 (>0.999)
Negative Correlation Weights	1.380 (0.248)	–0.212 (0.040)	–0.215 (0.047)	–0.208 (0.045)	–0.203 (0.047)	*n/a*					
System Segregation	**4.088 (0.007)**	0.129 (0.239)	0.028 (0.224)	0.093 (0.223)	0.041 (0.019)	**0.101 (0.037)**	0.036 (>0.999)	**0.089 (0.019)**	−0.065 (0.404)	−0.013 (>0.999)	0.052 (0.385)
Participation Coefficient	**4.735 (0.003)**	0.684 (0.050)	0.670 (0.055)	0.693 (0.056)	0.700 (0.047)	0.014 (0.616)	−0.009 (>0.999)	−0.015 (0.189)	**−0.023 (0.038)**	**−0.029 (0.002)**	−0.007 (>0.999)
Modularity	2.590 (0.052)	0.457 (0.060)	0.438 (0.055)	0.462 (0.056)	0.452 (0.059)	*n/a*					
Local Efficiency	**3.363 (0.019)**	0.355 (0.060)	0.355 (0.062)	0.340 (0.056)	0.333 (0.059)	0.000 (>0.999)	0.015 (0.403)	**0.022 (0.039)**	0.015 (0.685)	0.022 (0.112)	0.007 (>0.999)
Global Efficiency	1.980 (0.116)	0.225 (0.020)	0.218 (0.023)	0.218 (0.022)	0.220 (0.024)	*n/a*					

*All *p*-vales for *post hoc* tests are Bonferroni-corrected, with bolded values reflecting *p* < 0.05 after correction, and italicized values reflecting marginal differences (*p* < 0.10 after correction).*

**TABLE 3:** Interactions between age and correlation direction (main effect of age row), and age and network (rows depicting network effects), with accompanying post hoc tests of the group mean differences (*p*-values in parentheses) among all age group combinations.

**Direction**	**Network**	***F* (*p*-value)**	***Post Hoc* Tests**						**Corr w Age (*p*-value)**
			**YA vs. yMA**	**YA vs. oMA**	**YA vs. OA**	**yMA vs. oMA**	**yMA vs. OA**	**oMA vs. OA**	
Within	Main Effect of Age	** 12.813 (<0.001)**	0.007 (>0.999)	**0.024 (<0.001)**	**0.034 (<0.001)**	*0.017 (0.086)*	** 0.027 (0.001)**	0.010 (0.482)	**−0.163 (0.001)**
	Hand	2.289 (0.078)	*n/a*						
	Mouth	**5.220 (0.002)**	0.038 (0.397)	**0.047 (0.037)**	** 0.065 (0.001)**	0.009 (>0.999)	0.027 (>0.999)	0.018 (>0.999)	**−0.182 (<0.001)**
	Vis	2.321 (0.075)	*n/a*						
	Aud	** 10.240 (<0.001)**	−0.008 (>0.999)	**0.041 (0.005)**	**0.051 (<0.001)**	** 0.049 (0.004)**	**0.059 (<0.001)**	0.011 (>0.999)	**−0.254 (<0.001)**
	DMN	0.819 (0.484)	*n/a*						
	FP	0.625 (0.599)	*n/a*						
	VAN	1.247 (0.292)	*n/a*						
	CO	**9.187 (<0.001)**	0.034 (0.164)	**0.047 (0.001)**	**0.063 (<0.001)**	0.013 (>0.999)	0.029 (0.257)	0.016 (0.944)	**−0.246 (<0.001)**
	DAN	** 10.904 (<0.001)**	0.021 (>0.999)	**0.068 (<0.001)**	**0.070 (<0.001)**	** 0.046 (0.040)**	**0.048 (0.024)**	0.002 (>0.999)	**−0.275 (<0.001)**
	Sal	1.577 (0.194)	*n/a*						
Between	Main Effect of Age	**3.943 (0.009)**	−0.005 (>0.999)	0.013 (0.244)	* 0.016 (0.079)*	0.018 (0.106)	**0.021 (0.036)**	0.003 (>0.999)	**−0.100 (0.039)**
	Hand	2.515 (0.058)	*n/a*						
	Mouth	1.117 (0.342)	*n/a*						
	Vis	1.365 (0.253)	*n/a*						
	Aud	1.899 (0.129)	*n/a*						
	DMN	1.887 (0.131)	*n/a*						
	FP	1.637 (0.180)	*n/a*						
	VAN	1.817 (0.143)	*n/a*						
	CO	**4.672 (0.003)**	−0.008 (>0.999)	0.010 (0.866)	** 0.019 (0.042)**	0.019 (0.151)	**0.027 (0.006)**	0.008 (>0.999)	**−0.151 (0.002)**
	DAN	**3.645 (0.013)**	−0.011 (>0.999)	0.013 (0.642)	0.015 (0.316)	*0.025 (0.059)*	**0.027 (0.025)**	0.002 (>0.999)	**−0.119 (0.014)**
	Sal	**4.078 (0.007)**	0.000 (>0.999)	0.012 (0.413)	** 0.020 (0.015)**	0.012 (0.676)	*0.020 (0.056)*	0.008 (>0.999)	**−0.165 (0.001)**

**F*-values reflect the main effect of age for each network/metric, and mean differences reflect the comparisons among groups driving the significance of the main effect. The final column also presents the correlation between each connectivity metric and the continuous metric of age (Pearson correlation coefficient, with *p*-value in parentheses) for each metric showing a significant effect of age group. All *p*-vales for *post hoc* tests are Bonferroni-corrected, with bolded values reflecting *p* < 0.05 after correction, and italicized values reflecting marginal differences (*p* < 0.10 after correction).*

**FIGURE 5:**
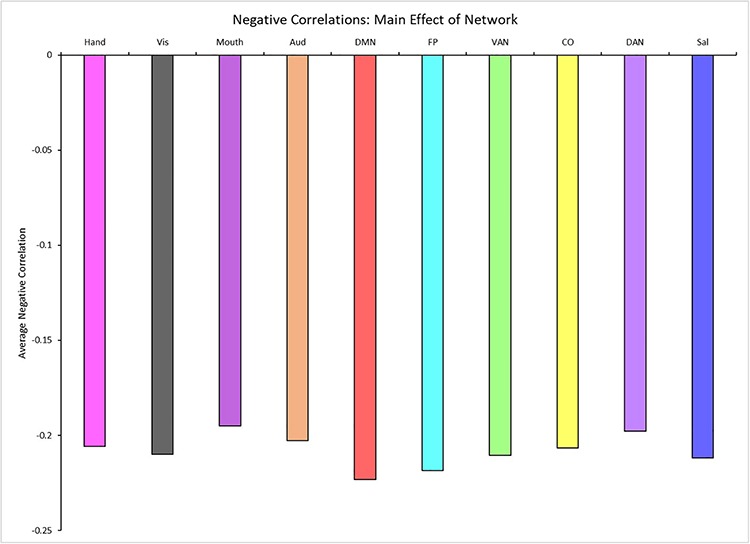
Main effect of network showing the average negative between-network correlation for each network (for significant differences see Table 4). Network colors correspond to their color assignments in Figures 1–3.

A 4 (age group: YA, yMA, oMA, OA) × 10 (network) MANCOVA revealed a significant effect of network (*F*_9__,__3798_ = 19.193, *p* < 0.001) on negative between-network correlation weight. The main effect of age group (*F*_3__,__422_ = 1.380, *p* = 0.248), and the interaction between network and age group (*F*_27__,__3798_ = 0.861, *p* = 0.672) were not significant. Follow-up analyses probing the main effect of network showed that some networks showed stronger between-network negative correlations than others (see Table 4 and Figure 5).

**TABLE 4:** Mean differences among all possible network combinations (*p*-values in parentheses) for between-network negative correlations (averaged across all participants), depicting the results of the main effect of network on the magnitude of between-network negative correlations.

**Network**	**Hand**	**Vis**	**Mouth**	**Aud**	**DMN**	**FP**	**VAN**	**CO**	**DAN**	**Sal**
Hand	*n/a*	0.003 (>0.999)	**−0.012 (0.002)**	−0.004 (>0.999)	**0.016 (<0.001)**	**0.012 (<0.001)**	0.003 (>0.999)	0.000 (>0.999)	**−0.008 (0.004)**	0.004 (>0.999)
Vis	0.003 (>0.999)	*n/a*	**−0.015 (<0.001)**	−0.007 (0.092)	**0.013 (<0.001)**	**0.009 (0.003)**	0.000 (>0.999)	−0.003 (>0.999)	**−0.011 (<0.001)**	0.001 (>0.999)
Mouth	**−0.012 (0.002)**	**−0.015 (<0.001)**	*n/a*	0.007 (0.491)	**0.028 (<0.001)**	**0.024 (<0.001)**	**0.015 (<0.001)**	**0.012 (0.002)**	0.004 (>0.999)	**0.016 (<0.001)**
Aud	−0.004 (>0.999)	−0.007 (0.092)	0.007 (0.491)	*n/a*	**0.021 (<0.001)**	**0.017 (<0.001)**	**0.008 (0.040)**	0.005 (>0.999)	−0.004 (>0.999)	**0.009 (0.008)**
DMN	**0.016 (<0.001)**	**0.013 (<0.001)**	**0.028 (<0.001)**	**0.021 (<0.001)**	*n/a*	−0.004 (0.577)	**−0.013 (<0.001)**	**−0.016 (<0.001)**	**−0.024 (<0.001)**	**−0.012 (<0.001)**
FP	**0.012 (<0.001)**	**0.009 (0.003)**	**0.024 (<0.001)**	**0.017 (<0.001)**	−0.004 (0.577)	*n/a*	**−0.009 (0.002)**	**−0.012 (<0.001)**	**−0.02 (<0.001)**	**−0.008 (<0.001)**
VAN	0.003 (>0.999)	0.000 (>0.999)	**0.015 (<0.001)**	**0.008 (0.040)**	**−0.013 (<0.001)**	**−0.009 (0.002)**	*n/a*	−0.003 (>0.999)	**−0.011 (<0.001)**	0.001 (>0.999)
CO	0.000 (>0.999)	−0.003 (>0.999)	**0.012 (0.002)**	0.005 (>0.999)	**−0.016 (<0.001)**	**−0.012 (<0.001)**	−0.003 (>0.999)	*n/a*	**−0.008 (0.010)**	0.004 (>0.999)
DAN	**−0.008 (0.004)**	**−0.011 (<0.001)**	0.004 (>0.999)	−0.004 (>0.999)	**−0.024 (<0.001)**	**−0.02 (<0.001)**	**−0.011 (<0.001)**	**−0.008 (0.010)**	*n/a*	**0.012 (<0.001)**
Sal	0.004 (>0.999)	0.001 (>0.999)	**0.016 (<0.001)**	**0.009 (0.008)**	**−0.012 (<0.001)**	**−0.008 (<0.001)**	0.001 (>0.999)	0.004 (>0.999)	**0.012 (<0.001)**	*n/a*

*The table is symmetrical along the diagonal in order to better illustrate patterns of significant differences for each network. All *p*-vales are Bonferroni-corrected, with bolded values reflecting *p* < 0.05 after correction.*

A one-way ANOVA testing the effect of age group on system segregation showed that this metric significantly differed by age group (*F*_3__,__415_ = 4.088, *p* = 0.007). Bonferroni-corrected *post hoc* analyses revealed that this effect of age was driven by greater system segregation in YAs compared to yMAs and OAs (see Table 2). Further, the continuous effect of age on system segregation was significant (*r*_426_ = −0.119, *p* = 0.014), such that higher age was associated with reduced system segregation.

### Participation Coefficient and Modularity

**FIGURE 6:**
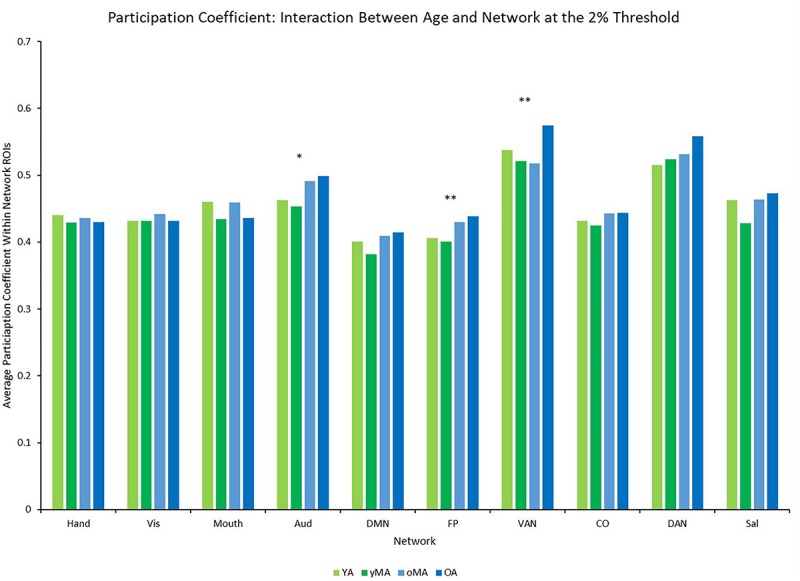
Average Participation Coefficient (PC) by network for each age group (light green = young adults, darker green = younger middle adults, light blue = older middle adults, darker blue = older adults). Significance of the main effect of age at each network is represented by asterisks: ^∗∗^*p* < 0.01, ^∗^*p* < 0.05.

A 4 (age group: YA, yMA, oMA, OA) × 9 (threshold: 2–10%) × 10 (network) MANCOVA revealed significant effects of age group (*F*_3__,__422_ = 4.735, *p* = 0.003), threshold (*F*_8__,__3376_ = 8824.174, *p* < 0.001), and network (*F*_9__,__3798_ = 69.835, *p* < 0.001) on participation coefficient. Additionally, interactions among threshold and network (*F*_72__,__30384_ = 23.173, *p* < 0.001), and the three-way interaction (*F*_216__,__30384_ = 1.615, *p* < 0.001) were significant, however, the interactions between threshold and age group (*F*_24__,__3376_ = 1.433, *p* = 0.079), and between network and age group (*F*_27__,__3798_ = 1.182, *p* = 0.237) were not significant. The main effect of age was driven by lower participation coefficient in yMAs compared to oMAs and OAs (see Table 2). Follow-up analyses of the three-way interaction showed that there was only a significant interaction between network and age group at the 2% threshold (*F*_27__,__3798_ = 1.751, *p* = 0.010), such that YAs and yMAs showed lower participation coefficient than OAs in the FP network, and oMAs showed lower participation coefficient than OAs in the VAN (see Table 5 and Figure 6). Further, correlational analyses revealed a linear of age on participation coefficient in the auditory and fronto-parietal networks, such that higher age was associated with greater network-level participation coefficient (see Table 5).

**TABLE 5:** Mean differences between groups (*p*-values in parentheses) probing the Participation Coefficient and Local Efficiency interactions among age and network, depicted here at the 2% threshold.

**Metric**	**Network**	***F* (*p*-value)**	***Post Hoc* Tests**						**Corr w Age (*p*-value)**
			**YA vs. yMA**	**YA vs. oMA**	**YA vs. OA**	**yMA vs. oMA**	**yMA vs. OA**	**oMA vs. OA**	
PC	Hand	0.209 (0.890)	*n/a*						
	Vis	0.626 (0.599)	*n/a*						
	Mouth	0.681 (0.564)	*n/a*						
	Aud	**3.346 (0.019)**	0.009 (>0.999)	−0.031 (0.386)	−0.038 (0.105)	−0.039 (0.246)	*−0.047 (0.077)*	−0.008 (>0.999)	**0.135 (0.005)**
	DMN	2.023 (0.110)	*n/a*						
	FP	**4.360 (0.005)**	0.004 (>0.999)	−0.026 (0.221)	−**0.035 (0.022)**	−0.030 (0.233)	−**0.039 (0.035)**	−0.009 (>0.999)	**0.165 (0.001)**
	VAN	**4.735 (0.003)**	0.018 (>0.999)	0.023 (>0.999)	−0.032 (0.326)	0.005 (>0.999)	−0.051 (0.061)	−**0.056 (0.003)**	0.085 (0.079)
	CO	0.599 (0.616)	*n/a*						
	DAN	1.970 (0.118)	*n/a*						
	Sal	2.494 (0.060)	*n/a*						
LE	Hand	**4.694 (0.003)**	0.003 (>0.999)	0.020 (0.175)	**0.031 (0.005)**	0.017 (0.672)	0.028 (0.061)	0.010 (>0.999)	−**0.185 (<0.001)**
	Vis	**3.434 (0.017)**	−0.013 (>0.999)	−0.001 (>0.999)	0.022 (0.214)	0.012 (>0.999)	**0.035 (0.030)**	0.023 (0.129)	−**0.102 (0.035)**
	Mouth	2.070 (0.104)	*n/a*						
	Aud	0.033 (0.992)	*n/a*						
	DMN	0.564 (0.639)	*n/a*						
	FP	0.542 (0.654)	*n/a*						
	VAN	**2.875 (0.036)**	0.035 (0.444)	**0.046 (0.025)**	0.025 (0.728)	0.011 (>0.999)	−0.011 (>0.999)	−0.022 (0.849)	−0.082 (0.092)
	CO	1.801 (0.146)	*n/a*						
	DAN	2.066 (0.104)	*n/a*						
	Sal	0.385 (0.764)	*n/a*						

**F*-values reflect the main effect of age for each network, and mean differences reflect the comparisons among groups driving the significance of the main effect. The final column also presents the correlation between each connectivity metric and the continuous metric of age (Pearson correlation coefficient, with *p*-value in parentheses) for each metric showing a significant effect of age group. All *p*-vales for *post hoc* tests are Bonferroni-corrected, with bolded values reflecting *p* < 0.05 after correction, and italicized values reflecting marginal differences (*p* < 0.10 after correction).*

A 4 (age group: YA, yMA, oMA, OA) × 9 (threshold: 2–10%) MANCOVA revealed a significant effect of threshold (*F*_8__,__3376_ = 2475.356, *p* < 0.001) and a significant interaction between threshold and age group (*F*_24__,__3376_ = 1.760, *p* = 0.013) on modularity, however, no significant main effect of age group (*F*_3__,__422_ = 2.590, *p* = 0.052). Follow-up analyses of the interaction between threshold and age group showed that oMAs showed greater modularity than yMAs at thresholds of 5–8% and 10% (*p*-values for the mean difference ranging from 0.020 to 0.040).

### Local and Global Efficiency

**FIGURE 7:**
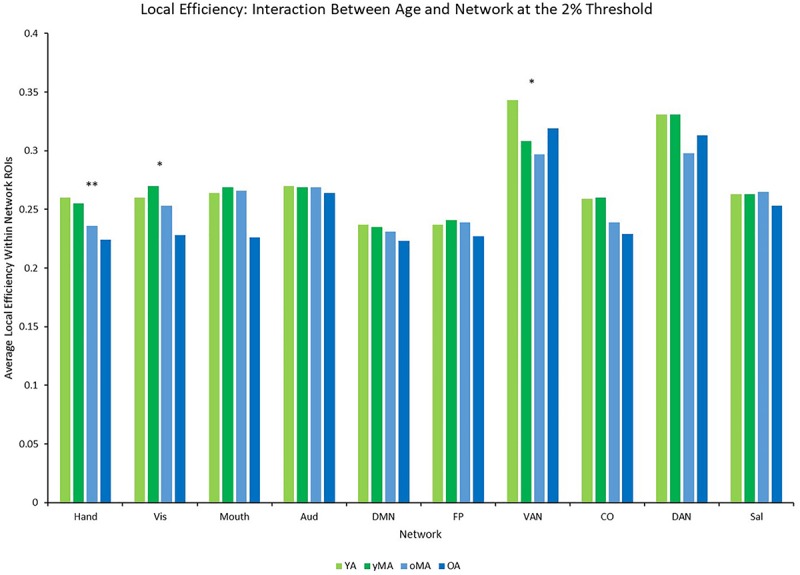
Average Local Efficiency (LE) by network for each age group (light green = young adults, darker green = younger middle adults, light blue = older middle adults, darker blue = older adults). Significance of the main effect of age at each network is represented by asterisks: ^∗∗^*p* < 0.01, ^∗^*p* < 0.05.

A 4 (age group: YA, yMA, oMA, OA) × 9 (threshold: 2–10%) × 10 (network) MANCOVA revealed significant effects of age group (*F*_3__,__422_ = 3.363, *p* = 0.019), threshold (*F*_8__,__3376_ = 1766.923, *p* < 0.001), and network (*F*_9__,__3798_ = 40.658, *p* < 0.001) on local efficiency. Additionally interactions among threshold and network (*F*_72__,__30384_ = 16.085, *p* < 0.001), and the three-way interaction (*F*_216__,__30384_ = 1.291, *p* = 0.003) were significant, while the interactions between network and age group (*F*_27__,__3798_ = 0.912, *p* = 0.595), and age group and threshold (*F*_24__,__3376_ = 0.714, *p* = 0.842) were not. This main effect of age was driven by lower local efficiency in OAs compared to YAs (see Table 2). Follow-up analyses of the three-way interaction showed that there was only a significant interaction between network and age group at the 2% threshold (*F*_27__,__3798_ = 1.592, *p* = 0.027) in the Hand (YA > OA), Vis (yMA > OA), and VAN (YA > oMA) networks (see Table 5 and Figure 7). Further, both linear effects of age on local efficiency were significant for the Hand and Vis networks, but not for the VAN network (see Table 5).

A 4 (age group: YA, yMA, oMA, OA) × 9 (threshold: 2–10%) MANCOVA revealed a significant effect of threshold (*F*_8__,__3376_ = 3191.555, *p* < 0.001) and a significant interaction between threshold and age group (*F*_24__,__3384_ = 3.337, *p* < 0.001) on global efficiency, however, no significant main effect of age group (*F*_3__,__423_ = 1.980, *p* = 0.116). Follow-up analyses of the interaction between threshold and age group showed that global efficiency only marginally differed by age group at a threshold of 3% (*F*_3__,__426_ = 2.471, *p* = 0.061), and that this was driven by marginally greater global efficiency in OAs than yMAs (mean difference = 0.011, *p* = 0.065).

### Cognitive Correlates of Connectivity

**FIGURE 8:**
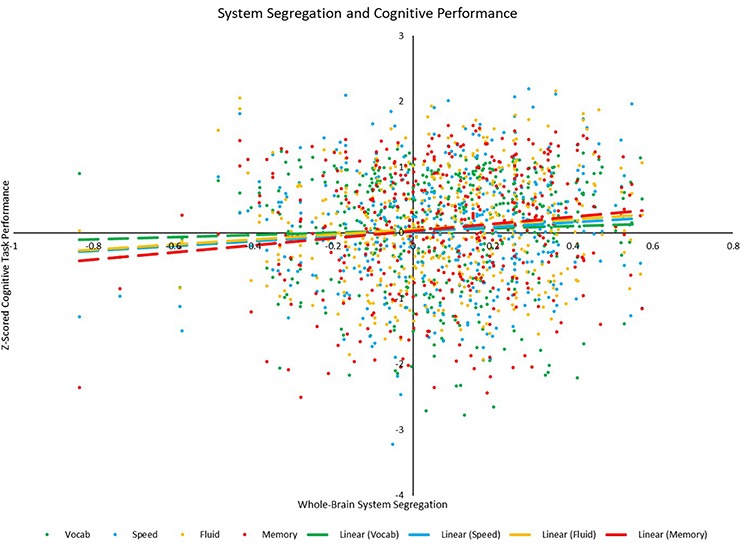
Scatterplot depicting the relationships between whole-brain system segregation and each of the four cognitive *z*-scores. Relationships between system segregation and speed (*p* = 0.047), fluid (*p* = 0.025), and memory (*p* = 0.007) were significant, while the relationship between system segregation and vocabulary (*p* = 0.366) was not.

**FIGURE 9:**
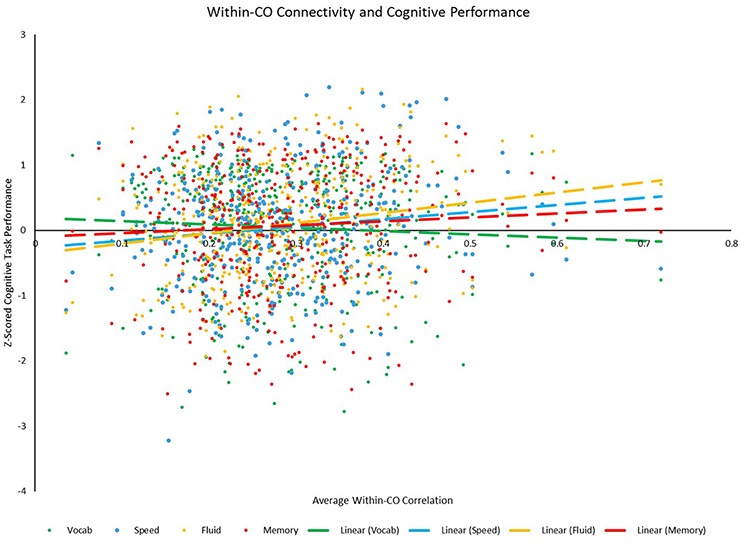
Scatterplot depicting the relationships between average positive within-CO correlation and each of the four cognitive *z*-scores. Relationships between within-CO correlation and speed (*p* = 0.011) and fluid (*p* < 0.001) were significant, while the relationships between within-CO correlation and vocabulary (*p* = 0.262) and memory (*p* = 0.201) were not.

Correlational analyses revealed several whole-brain and network-based metrics that were related to neuropsychological task performance outside of the scanner (see Table 6). For SPEED tasks, whole-brain system segregation (*r* = 0.097, *p* = 0.047; see Figure 8), and hand (positive between-network: *r* = 0.136, *p* = 0.006; LE: *r* = 0.099, *p* = 0.045), auditory (positive within-network: *r* = 0.151, *p* = 0.002; PC: *r* = −0.134, *p* = 0.006), CO (positive within-network: 0.125, *p* = 0.011; positive between-network: 0.113, *p* = 0.021; see Figure 9), and DAN (positive within-network: 0.136, *p* = 0.006; positive between-network: 0.121, *p* = 0.014) network integrity seemed to be related to task performance. For FLUID tasks, whole-brain system segregation (*r* = 0.110, *p* = 0.025), and Vis (positive within-network: *r* = 0.108, *p* = 0.027; negative between-network: *r* = −0.111, *p* = 0.024) and VAN (positive within-network: *r* = 0.187, *p* < 0.001; positive between-network: 0.114, *p* = 0.020; LE: *r* = 0.122, *p* = 0.013) network integrity was related to task performance. For MEM tasks, whole-brain system segregation was related to memory task performance (*r* = 0.134, *p* = 0.007), however, no network showed a consistent relationship between functional connectivity and task performance. Additionally, correlational analyses were conducted between VOCAB task performance and functional connectivity metrics, however, given the superior performance on these tasks by OAs (who show reduced network structure/integrity based on many of the analyses presented above), these trends are less interpretable, and thus will not be discussed here.

**TABLE 6:** Pearson correlation coefficients (*p*-values in parentheses) between each connectivity metric and *z*-scores of performance on Neuropsychological tasks falling into four domains: vocabulary (VOCAB), processing speed (SPEED), fluid reasoning (FLUID), and episodic memory (MEM).

**Connectivity Metric**	**Whole Brain**	**Network**									
		**Hand**	**Vis**	**Mouth**	**Aud**	**DMN**	**FP**	**VAN**	**CO**	**DAN**	**Sal**
**VOCAB**											
Within-network positive	*n/a*	−**0.129 (0.008)**	−0.037 (0.457)	−0.002 (0.974)	−0.096 (0.050)	−0.009 (0.859)	0.003 (0.947)	−0.016 (0.743)	−0.055 (0.262)	−**0.164 (0.001)**	−0.024 (0.624)
Between-network positive	*n/a*	−0.093 (0.059)	−**0.104 (0.034)**	−0.062 (0.208)	−**0.137 (0.005)**	−**0.128 (0.009)**	−0.096 (0.051)	−**0.103 (0.035)**	−**0.111 (0.024)**	−**0.126 (0.010)**	−0.078 (0.114)
Between-network negative	*n/a*	0.096 (0.050)	0.022 (0.659)	0.071 (0.147)	0.075 (0.126)	0.051 (0.298)	**0.107 (0.029)**	**0.114 (0.021)**	0.062 (0.212)	**0.108 (0.028)**	0.047 (0.336)
System Segregation	0.045 (0.366)	*n/a*									
Participation Coefficient (2%)	0.015 (0.765)	−0.024 (0.625)	−0.089 (0.069)	0.032 (0.513)	0.021 (0.664)	0.075 (0.127)	0.014 (0.771)	0.032 (0.518)	0.002 (0.962)	0.027 (0.581)	−0.019 (0.704)
Modularity (10%)	**0.097 (0.049)**	*n/a*									
Local Efficiency (2%)	−0.067 (0.173)	−0.096 (0.051)	−**0.104 (0.034)**	0.018 (0.707)	0.019 (0.693)	0.001 (0.990)	−0.062 (0.206)	−0.019 (0.700)	0.000 (0.994)	−0.032 (0.519)	−0.053 (0.280)
Global Efficiency (3%)	−0.001 (0.978)	*n/a*									
**SPEED**											
Within-network positive	*n/a*	0.047 (0.337)	0.087 (0.077)	**0.191 (<0.001)**	**0.151 (0.002)**	0.090 (0.068)	0.034 (0.492)	−0.022 (0.660)	**0.125 (0.011)**	**0.136 (0.006)**	0.043 (0.387)
Between-network positive	*n/a*	**0.136 (0.006)**	0.082 (0.095)	0.059 (0.228)	0.086 (0.080)	0.073 (0.137)	0.073 (0.136)	0.077 (0.116)	**0.113^ (0.021)**	**0.121 (0.014)**	**0.128 (0.009)**
Between-network negative	*n/a*	−0.067 (0.172)	−0.066 (0.182)	−0.074 (0.134)	−0.045 (0.362)	−0.057 (0.251)	−0.034 (0.490)	0.001 (0.982)	−0.062 (0.207)	−0.015 (0.764)	−0.026 (0.601)
System Segregation	**0.097 (0.047)**	*n/a*									
Participation Coefficient (2%)	−0.071 (0.152)	0.011 (0.831)	−0.031 (0.524)	−0.004 (0.932)	−**0.134 (0.006)**	−0.068 (0.169)	−0.038 (0.437)	−0.040 (0.420)	−0.052 (0.295)	−0.077 (0.118)	−0.001 (0.982)
Modularity (10%)	0.036 (0.463)	*n/a*									
Local Efficiency (2%)	0.085 (0.085)	**0.099 (0.045)**	0.061 (0.219)	0.014 (0.771)	−0.028 (0.576)	0.024 (0.624)	0.082 (0.095)	0.016 (0.752)	0.073 (0.135)	0.043 (0.385)	0.074 (0.131)
Global Efficiency (3%)	−0.024 (0.632)	*n/a*									
**FLUID**											
Within-network positive	*n/a*	0.051 (0.300)	**0.108 (0.027)**	**0.143 (0.004)**	**0.165 (0.001)**	0.066 (0.182)	0.015 (0.755)	0.036 (0.465)	**0.187 (<0.001)**	0.087 (0.078)	0.020 (0.688)
Between-network positive	*n/a*	0.083 (0.090)	0.006 (0.900)	0.011 (0.818)	0.080 (0.106)	0.032 (0.515)	0.058 (0.240)	0.055 (0.263)	**0.114^ (0.020)**	0.064 (0.193)	**0.116 (0.018)**
Between-network negative	*n/a*	−0.095 (0.052)	−**0.111 (0.024)**	−0.018 (0.717)	−0.037 (0.447)	−0.067 (0.174)	−0.067 (0.171)	0.015 (0.759)	−0.092 (0.061)	−0.040 (0.422)	−0.031 (0.524)
System Segregation	**0.110 (0.025)**	*n/a*									
Participation Coefficient (2%)	−0.028 (0.564)	0.064 (0.194)	−0.023 (0.643)	0.024 (0.630)	−0.063 (0.204)	0.016 (0.744)	−0.087 (0.076)	0.009 (0.848)	−0.019 (0.707)	−0.087 (0.075)	−0.075 (0.130)
Modularity (10%)	0.058 (0.242)	*n/a*									
Local Efficiency (2%)	0.091 (0.066)	**0.135 (0.006)**	0.038 (0.443)	0.028 (0.575)	0.062 (0.211)	0.059 (0.232)	−0.023 (0.643)	**0.097 (0.049)**	**0.122^ (0.013)**	0.039 (0.428)	−0.028 (0.575)
Global Efficiency (3%)	0.060 (0.221)	*n/a*									
**MEM**											
Within-network positive	*n/a*	0.026 (0.600)	0.065 (0.186)	**0.162 (0.001)**	**0.118 (0.017)**	0.059 (0.235)	−0.075 (0.128)	−**0.102 (0.038)**	0.063 (0.201)	**0.142 (0.004)**	**0.098^ (0.048)**
Between-network positive	*n/a*	0.082 (0.098)	0.014 (0.771)	0.006 (0.909)	0.077 (0.118)	0.046 (0.356)	0.011 (0.828)	0.062 (0.211)	0.091 (0.065)	0.060 (0.228)	0.070 (0.158)
Between-network negative	*n/a*	−0.013 (0.796)	−0.074 (0.134)	−0.023 (0.646)	−0.012 (0.816)	0.007 (0.885)	−0.018 (0.718)	0.013 (0.786)	−0.015 (0.755)	−0.015 (0.763)	0.047 (0.346)
System Segregation	**0.134 (0.007)**	*n/a*									
Participation Coefficient (2%)	−0.044 (0.370)	0.006 (0.908)	−0.014 (0.783)	0.037 (0.459)	−0.048 (0.333)	−0.059 (0.234)	−0.057 (0.247)	−0.071 (0.151)	0.042 (0.394)	−0.061 (0.218)	0.000 (>0.999)
Modularity (10%)	0.024 (0.631)	*n/a*									
Local Efficiency (2%)	0.063 (0.204)	0.058 (0.244)	0.076 (0.123)	0.009 (0.854)	0.026 (0.596)	0.011 (0.829)	0.045 (0.360)	0.001 (0.988)	0.095 (0.054)	0.022 (0.657)	0.009 (0.851)
Global Efficiency (3%)	−0.025 (0.612)	*n/a*									

*Correlations marked with “ **^** ” denote correlations whose *p*-values rise above 0.500 when correlational analyses are conducted on only those participants who completed the longer, 9.5-min resting state scan; all other significant correlations either remain significant, or have *p*-values that are between 0.050 and 0.500. No correction for multiple comparisons was performed; bolded values reflect *p*-values less than 0.05.*

### Effect of Scan Length on Analyses

Due to the differences in resting state scan length, analyses were repeated including a factor representing the length of the resting state scan (short rest scan *N* = 141: YA *n* = 40, yMA *n* = 20, oMA *n* = 37, OA *n* = 44; long rest scan *N* = 286: YA *n* = 61, yMA *n* = 41, oMA *n* = 89, OA *n* = 95). Specifically, the primary focus of these analyses was to assess whether the primary effects of age on connectivity observed above were affected by the length of the resting state scan. As such, the primary interactions of interest were: (1) positive correlations: Network × Direction × Age Group × Scan Length; (2) system segregation: Age Group × Scan Length; (3) participation coefficient: Network × Threshold × Age Group × Scan Length; (4) modularity: Threshold × Age Group × Scan Length; (5) local efficiency: Network × Threshold × Age Group × Scan Length; (6) correlations: all correlations showing significant relationships between connectivity metrics and cognitive performance. Results of these specific tests showed: (1) positive correlations: no modification of this three-way interaction as a function of scan length (*F*_27__,__3744_ = 0.978, *p* = 0.497); (2) system segregation: no interaction between age group and scan length (*F*_3__,__418_ = 1.037, *p* = 0.376); (3) participation coefficient: no modification of this three-way interaction as a function of scan length (*F*_216__,__30096_ = 0.775, *p* = 0.994); (4) modularity: significant interaction among Threshold, Age Group, and Scan Length (*F*_24__,__3344_ = 2.316, *p* < 0.001); (5) local efficiency: significant interaction among Network, Threshold, Age Group, and Scan Length (*F*_216__,__30096_ = 1.235, *p* = 0.011); (6) correlations: Four correlations showing considerable change in *p*-value when re-analyzed in just those participants who completed a longer resting state scan.

For the metric of modularity, the effect of scan length on the interaction between threshold and age group showed that there was a marginally significant interaction between age group and threshold for participants who completed the longer rest scan (*F*_24__,__2248_ = 1.534, *p* = 0.047), but there was a strong, significant interaction between age group and threshold for those who completed the shorter rest scan (*F*_24__,__1088_ = 2.734, *p* < 0.001). However, *post hoc* tests for the short rest scan, revealed only a marginal effect of age group on modularity at the 2% threshold, driven by marginal differences between younger middle adults and older middle (mean difference = −0.043, *p* = 0.150) and older (mean difference = −0.041, *p* = 0.160) adults. For comparison, analysis of modularity in the full sample revealed a significant difference between younger middle and older middle adults at thresholds 5–8 and 10%.

For the metric of local efficiency, the effect of scan length on the interaction among network, threshold, and age group showed that there was a marginally significant interaction among network, threshold, and age group for participants who completed the longer rest scan (*F*_72__,__20232_ = 1.178, *p* = 0.038), but there was a stronger, significant interaction between age group and threshold for those who completed the shorter rest scan (*F*_72__,__9792_ = 1.223, *p* = 0.015). *Post hoc* tests for the shorter resting state scan showed a marginal interaction between network and age group at the 2% threshold (*F*_27__,__1224_ = 1.386, *p* = 0.091), but no significant interaction at any other threshold (all *p*-values > 0.615). This interaction at the 2% level showed a significant effect of age group in the visual (yMA > oMA, *p* = 0.020; yMA > OA, *p* = 0.020), mouth (yMA > OA, *p* = 0.010), and cingulo-opercular (yMA > oMA, *p* = 0.036; yMA > OA, *p* = 0.003) networks. *Post hoc* tests for the longer resting state scan showed a significant interaction between network and age group at the 2% threshold (*F*_27__,__2529_ = 1.503, *p* = 0.047), but no significant interaction at any other threshold (all *p*-values > 0.259). This interaction at the 2% level showed a significant effect of age group in the hand (YA > OA, *p* = 0.028), visual (oMA > OA, *p* = 0.014), ventral attention (YA > oMA, *p* = 0.026), and dorsal attention (YA > oMA, *p* = 0.039) networks. For comparison, analysis of local efficiency in the full sample revealed a significant effect of age at the 2% threshold within the hand (YA > OA), visual (yMA > OA), and ventral attention (YA > oMA) networks.

For the correlations between connectivity metrics and neuropsychological task performance, four correlations that showed significant relationships between connectivity metrics and task performance in the full sample showed *p*-values greater than 0.500 when analyses were conducted in just those participants who completed the longer resting state scan: positive between-CO and Speed (*r* = 0.025, *p* = 0.674), positive between-CO and Fluid (*r* = 0.011, *p* = 0.859), positive within-Salience and Memory (*r* = 0.019, *p* = 0.754), and 2% CO LE and Fluid (*r* = −0.030, *p* = 0.624).

## Discussion

Results from the present study suggest that age has an effect on several whole-brain metrics of functional connectivity. While several past studies have noted effects of aging on modularity (Betzel et al., 2014; Song et al., 2014; Geerligs et al., 2015), and between-network connections (Betzel et al., 2014; Chan et al., 2014) at rest, the present study did not find a similar effect of aging on these metrics. However, the present study did replicate past studies finding an increase in participation coefficient (Chan et al., 2014; Geerligs et al., 2015), and reductions in within-network connectivity (Betzel et al., 2014; Chan et al., 2014; Geerligs et al., 2015), system segregation (Chan et al., 2014), and local efficiency (Song et al., 2014; Geerligs et al., 2015) across the adult age range.

Of note, however, is that while a past study examining the effect of age on system segregation found that this was driven by both decreasing within-network connections and increasing between-network connections (Chan et al., 2014), the present study found that this decrease in system segregation was primarily driven by reductions in within-network connectivity. While results did show an effect of age on between-network correlations, the relationship between age and between-network correlations was negative (not positive as in Chan et al., 2014), and this was driven by reduced between-network correlation strength in OAs relative to yMAs (and not younger adults). Further, the correlation between between-network connectivity and age was quite weak, and possibly localized to a negative relationship between age and between-network connectivity for the CO and Sal networks. Some key differences between the present study and that by Chan et al. (2014), however, may explain some of this difference: (1) the present study utilized the Power et al. (2011) network parcellation scheme rather than defining networks based on participants’ optimal network organization, (2) the present study used the Power et al. (2011) 264-ROI parcellation scheme, while Chan et al. (2014) used a novel 441-ROI parcellation scheme, and (3) the present study did not include global signal regression in the processing pipeline. While Chan et al. (2014) did cross-reference their own network parcellation with that of Power et al. (2011), slight differences in ROI number, location, and network assignment may have contributed to this difference in the observed patterns of results. Thus, while this reduction in system segregation may be driven by declining within-network connectivity alongside increasing between-network connectivity (as in Chan et al., 2014), it may be the case that declining within-network connectivity is more readily and consistently reproduced across different network parcellations and processing pipelines (as in the present study).

The present study also examined several of the metrics described above at the network-level in order to probe which networks might be specifically susceptible to age-related decline. Past studies found increases in connectivity with age within the somatomotor (Tomasi and Volkow, 2012; Song et al., 2014) and auditory (King et al., 2017) networks, and decreases in connectivity with age within the visual (Betzel et al., 2014), default mode (Andrews-Hanna et al., 2007; Wang et al., 2010; Onoda et al., 2012; Tomasi and Volkow, 2012; Betzel et al., 2014; Song et al., 2014; Geerligs et al., 2015), fronto-parietal (Campbell et al., 2012; Betzel et al., 2014; Geerligs et al., 2015), cingulo-opercular (Geerligs et al., 2015), dorsal attention (Tomasi and Volkow, 2012), and salience (Onoda et al., 2012) networks. Findings from the present study provided support for age-related reductions in within-network connectivity in the cingulo-opercular and dorsal attention networks, however, contrary to previous studies, the present study also found decreases in within-network connectivity in the somatomotor (mouth) and auditory networks.

In addition to examining average within- and between-network connectivity in both the positive and negative directions on the network level, the present study also calculated two local graph theory metrics at the network level: participation coefficient and local efficiency. A previous study by Geerligs et al. (2015) found increases in participation coefficient localized to the visual and somatomotor networks, and reductions in local efficiency in aging localized to the cingulo-opercular, fronto-parietal, and default mode networks. Results from the present study provide some support for these trends, but fall short of replicating them completely: participation coefficient was found to be elevated in OAs (relative to yMAs and YAs, respectively) in the auditory and fronto-parietal networks, however local efficiency was only found to be reduced in OAs (relative to YAs) in the somatomotor hand network (and relative to yMAs in the visual network). While some of these findings seem to corroborate those of Geerligs et al. (2015), there are some key differences in the patterns observed. Comparisons to this study come with the same caveats as those with the Chan et al. (2014) study, but also with a key difference in samples: the Geerligs et al. (2015) study included 40 younger adults and 40 OAs, and thus did not include participants in middle-adulthood. Thus, the present study may have also failed to replicate some of the effects they observed due to differences in the age distribution of the sample, as well as differences in the power to detect effects among 2 versus 4 groups.

The present study, thus builds on these two foundational studies examining functional connectivity in aging by including a sample that spans a wide age range (unlike Geerligs et al., 2015), and including a variety of local and global graph theory metrics of functional connectivity (unlike Chan et al., 2014). Further, while several recent studies have endeavored to similarly build on or replicate these foundational studies, the present one is the first to examine all of the above metrics within one large sample, and across such a wide age range. Previous studies examining these effects in aging have differed from the present study in the scope of functional connectivity metrics used (King et al., 2017), sample size (Iordan et al., 2017; King et al., 2017), or age range (Iordan et al., 2017; Zonneveld et al., 2019); thus, the present study is critical in furthering this line of investigation in a systematic way by bridging several methodologies in a large lifespan sample.

Results from this study also suggest that resting state functional connectivity in certain networks may be specifically related to cognitive function across the adult age range. At the whole-brain level, system segregation may be related to processing speed, fluid reasoning, and memory task performance, such that greater segregation is associated with better performance on the tasks. At the network level, integrity of the hand, auditory, and dorsal attention networks may be related to performance on processing speed tasks, integrity of the visual network may be related to performance on fluid reasoning tasks, and integrity of the cingulo-opercular network may be related to performance on both processing speed and fluid reasoning tasks. One interesting finding consistently observed across these functional connectivity correlates of cognitive task performance is that within-network positive connectivity, between-network positive connectivity, system segregation, and participation coefficient seem to show the strongest relationships with cognition. While past studies examining functional connectivity during a task seemed to focus on the role of between-network connections in explaining variability in task performance (Fox et al., 2005; Uddin et al., 2009; Grady et al., 2016; Varangis et al., 2019), results from the present study suggest that both within- and between-network resting state connectivity may also play a role in accounting for some of the variability in cognitive function across the adult lifespan. While these results are not seen as contradictory to those of these previous studies, they suggest that resting and task-based connectivity may exhibit differential relationships with cognitive performance, and thus the metrics of functional connectivity chosen for analysis may play a different role in resting vs. task-based conditions.

Studies examining more network-specific metrics of resting state connectivity have been somewhat mixed in finding relationships between network-based connectivity and task performance in the context of healthy aging. Five studies in particular examined relationships between within- and between-network connectivity and cognitive task performance: two studies implicated the DMN, one in verbal learning in just younger adults (CO-DMN and FP-DMN connections; Geerligs et al., 2015), and the other in fluid intelligence (Geerligs et al., 2017); one study implicated the FP network in working memory in just OAs (Geerligs et al., 2015); two studies implicated the CO network in working memory in just younger adults (CO-FP connections and LE in the CO, respectively; Geerligs et al., 2015; Iordan et al., 2017); and two studies implicated the Salience network, one in “frontal” processes (Onoda et al., 2012), and one in three different memory tasks (Sal between-network connectivity; La Corte et al., 2016). Further, Chan et al. (2014) found that system segregation (a metric derived from the within/between-network connectivity metrics discussed above) was related to associative memory performance. While the present study did not directly measure some of the cognitive domains included in these prior studies, some network-level trends were consistent with the results here – namely, that connectivity within the CO network, between the CO network and other networks, and local efficiency in the CO network may be related to tasks of executive function (here, fluid reasoning), that the salience network may be involved in tasks of executive function and memory, and that whole-brain system segregation may be related to memory performance. While some of these relationships between network-based connectivity and task performance may differ between our study and those previous studies, it should be noted that there are a few key differences between the present study and those mentioned above: (1) the present study includes differing cognitive measures that may not directly overlap with those utilized in previous studies, (2) the measure of between-network connectivity in the present study did not target specific network pairings, and (3) many of these relationships observed in prior studies were specific to older or younger adults and were not observed across the whole sample. As such, results from the present study do not necessarily provide support for or contradict results from prior studies, but rather add to the body of research showing that functional connectivity at rest can account for significant portions of the variability in out-of-scanner cognitive task performance.

These relationships between functional connectivity metrics and cognition in the context of aging suggest that functional brain organization, even at rest, may underlie some individual differences in cognitive function across the adult lifespan. This concept that functional brain architecture may support cognitive function in the face of age-related structural brain changes naturally evokes the concept of CR. The CR theory posits that exposures accrued throughout the lifespan (IQ, education, cognitive and social engagement, exercise, etc.) may buffer against some of the deleterious effects of structural brain changes on cognitive outcomes. The mechanism by which this is enacted is hotly debated, but is theorized to involve differential utilization of specific brain regions or networks by individuals with higher levels of CR. Recent studies have focused on identification of CR networks that may be utilized across multiple task and rest conditions, whose activation is correlated with IQ, and whose expression during a task moderates the effect of cortical thickness on task performance (Stern et al., 2018). The authors showed that individuals who demonstrated greater expression of this IQ-related CR network across multiple cognitive tasks showed a weaker relationship between cortical thickness and cognitive performance, suggesting that expression of this network played some role in dampening the effect of age-related cortical volume loss on cognition. While the present study does not relate the current findings to proxies of CR, it does suggest that some of the individual variability in cognitive functioning throughout adulthood can be accounted for by functional brain network properties. Future studies should therefore target the degree to which these relationships between functional connectivity and cognitive measures may be influenced by CR exposures, and whether these patterns of functional connectivity that are related to CR proxies may moderate the effect of volumetric differences between older and younger adults on cognitive outcomes.

### Limitations

While the present study benefited from a large sample and stringent fMRI processing criteria, there were several limitations that should be considered when interpreting results. First, participants in the present study completed two different lengths of resting state scan. A previous study assessing the effect of scan length on functional connectivity estimates found that test–retest reliability and similarity was optimized in resting state scans that were 9–12 min or longer (Birn et al., 2013). Thus, patterns observed in shorter resting state scans may not be as reliable or replicable within- or across participants. However, analyses testing whether this effect of scan length modified any of the significant effects of age observed above showed a relatively minor effect of scan length on the primary interactions discussed above. Specifically, effects of age on modularity and local efficiency may be slightly discounted since these metrics seem particularly sensitive to differences in scan length, and the four correlations that were found to be dramatically altered in the longer resting state scan sample might represent less reliable relationships between connectivity and cognition. Additionally, given that many of the age-related differences in modularity and local efficiency showed somewhat counter-intuitive patterns (i.e., differences between yMAs and oMAs), these differences were less interpretable and consistent. Of particular note is that the effects of age on within/between network positive correlations and system segregation were not affected by this difference in scan length, suggesting that these effects may be less sensitive to length of the resting state scan being analyzed.

Another potential limitation of the present study is the utilization of an externally derived network parcellation scheme for network assignment (Power et al., 2011). Several past studies have performed similar analyses using a network parcellation scheme derived from participants’ optimal network organization, and cross-registering these networks with nodal assignments in the Power et al. (2011) network taxonomy (Chan et al., 2014; Geerligs et al., 2015). While those studies benefit from deriving network assignments based on actual participant network structure, their results may be more difficult to reproduce in an external dataset due to possible differences in network structure in different samples. Several past studies have also utilized the Power et al. (2011) taxonomy to define network structure/organization, and thus indicate that this approach may be appropriate to estimate a plausible network structure that is not biased by participants in the sample (i.e., Song et al., 2014; Varangis et al., 2019). As such, both approaches may have advantages and disadvantages for examining whole-brain and network-based measures in the context of aging.

## Conclusion

We have shown that aging has an effect on several different metrics of functional connectivity at rest. Specifically, aging results in weakening within-network connectivity, lower system segregation and local efficiency, and higher participation coefficient. Further, the results suggest that nearly every primary sensory and cognitive network faces some degree of age-related decline, from reduced within-network connectivity (auditory, default mode, fronto-parietal, cingulo-opercular, dorsal attention, and salience networks), higher participation coefficient (somatomotor, visual, default mode, fronto-parietal, and ventral attention networks), or reduced local efficiency (visual network). Additionally, some of these connectivity metrics were related to cognitive performance. Altogether these results suggest a general reduction in network integrity in the context of aging, which could be associated with cognitive outcomes. These results also highlight the utility of a whole-brain, multi-technique approach to capturing different facets of functional connectivity that may be differentially sensitive to age-related decline in order to more completely encapsulate the effect of aging on large-scale brain connectivity.

## Data Availability

The datasets generated for this study are available on request to the corresponding author.

## Ethics Statement

The Columbia University Institutional Review Board approved all study procedures, and all participants provided written informed consent prior to the participation.

## Author Contributions

YS designed the parent studies and oversaw the data collection. QR preprocessed and prepared all the data. YS, CH, and QR consulted on analysis techniques and statistical methodology. EV analyzed the resting-state brain imaging and behavioral data, and wrote the original draft. All authors reviewed and edited the final manuscript.

## Conflict of Interest Statement

The authors declare that the research was conducted in the absence of any commercial or financial relationships that could be construed as a potential conflict of interest.

## Footnotes

1 www.brain-connectivity-toolbox.net
